# Evidence for a distinct neuro-immune signature in rats that develop behavioural disability after nerve injury

**DOI:** 10.1186/s12974-015-0318-4

**Published:** 2015-05-20

**Authors:** Paul J Austin, Annika M Berglund, Sherman Siu, Nathan T Fiore, Michelle B Gerke-Duncan, Suzanne L Ollerenshaw, Sarah-Jane Leigh, Priya A Kunjan, James WM Kang, Kevin A Keay

**Affiliations:** Discipline of Anatomy and Histology, School of Medical Sciences, The University of Sydney, Room E511, Anderson Stuart Building F13, Sydney, NSW 2006 Australia

**Keywords:** Sciatic nerve injury, Cytokine, T lymphocyte, Spinal cord, Social interactions, Behavioural disabilities, Affective-motivational disturbances

## Abstract

**Background:**

Chronic neuropathic pain is a neuro-immune disorder, characterised by allodynia, hyperalgesia and spontaneous pain, as well as debilitating affective-motivational disturbances (e.g., reduced social interactions, sleep-wake cycle disruption, anhedonia, and depression). The role of the immune system in altered sensation following nerve injury is well documented. However, its role in the development of affective-motivational disturbances remains largely unknown. Here, we aimed to characterise changes in the immune response at peripheral and spinal sites in a rat model of neuropathic pain and disability.

**Methods:**

Sixty-two rats underwent sciatic nerve chronic constriction injury (CCI) and were characterised as either *Pain and disability*, *Pain and transient disability* or *Pain alone* on the basis of sensory threshold testing and changes in post-CCI dominance behaviour in resident-intruder interactions. Nerve ultrastructure was assessed and the number of T lymphocytes and macrophages were quantified at the site of injury on day six post-CCI. ATF3 expression was quantified in the dorsal root ganglia (DRG). Using a multiplex assay, eight cytokines were quantified in the sciatic nerve, DRG and spinal cord.

**Results:**

All CCI rats displayed equal levels of mechanical allodynia, structural nerve damage, and reorganisation. All CCI rats had significant infiltration of macrophages and T lymphocytes to both the injury site and the DRG. *Pain and disability* rats had significantly greater numbers of T lymphocytes. CCI increased IL-6 and MCP-1 in the sciatic nerve. Examination of disability subgroups revealed increases in IL-6 and MCP-1 were restricted to *Pain and disability* rats. Conversely, CCI led to a decrease in IL-17, which was restricted to *Pain and transient disability* and *Pain alone* rats. CCI significantly increased IL-6 and MCP-1 in the DRG, with IL-6 restricted to *Pain and disability* rats. CCI rats had increased IL-1β, IL-6 and MCP-1 in the spinal cord. Amongst subgroups, only *Pain and disability* rats had increased IL-1β.

**Conclusions:**

This study has defined individual differences in the immune response at peripheral and spinal sites following CCI in rats. These changes correlated with the degree of disability. Our data suggest that individual immune signatures play a significant role in the different behavioural trajectories following nerve injury, and in some cases may lead to persistent affective-motivational disturbances.

## Background

It is now widely accepted that neuropathic pain is a neuro-immune disorder [1, 2]. Through their inflammatory mediators, immune and immune-like glial cells are able to directly activate and sensitise nociceptors, thereby increasing their excitability and contributing to central sensitisation in the dorsal horn of the spinal cord [1, 3, 4]. A major focus of research into the neuro-immune bases of pain has been the sensory-discriminative aspects of neuropathic pain (i.e. allodynia, hyperalgesia and spontaneous pain), primarily at peripheral and spinal cord sites. However, a role for the immune system in mediating the debilitating affective-motivational disturbances experienced by neuropathic pain patients, such as reduced familial and social interactions, sleep-wake cycle disruption, impaired cognition, reduced physical activity, lack of motivation, anhedonia and depression [5–17], are yet to be similarly investigated.

The idea that the immune system can regulate higher order behaviours has recently begun to gain traction. Individual differences in emotional coping styles are reflected in differences in the interactions of the immune system and the hypothalamic-pituitary-adrenal (HPA) axis, for detailed review see [18]. Furthermore, the neuro-immune interface may result in direct modulation of complex behaviours. For example, cytokines of peripheral origin act at multiple central nervous system (CNS) sites to trigger acute sickness behaviours (characterised by fatigue, reduced appetite and sleep-wake changes), as well as modulating depression and cognition [19, 20]. Recently, it has been suggested that high comorbidity of chronic pain and depression is indicative of a common mechanism involving chronic inflammation [21]. This view is supported by a study in the olfactory bulbectomised rat, where chronic treatment with systemic minocycline attenuated both depressive-like behaviour and nerve injury-induced allodynia, whilst concomitantly increasing the expression of anti-inflammatory markers in microglia of the prefrontal cortex [22]. There is also a growing number of studies in rodent models of neuropathic pain where peripheral nerve injury alone leads to increases in pro-inflammatory cytokines (e.g., interleukin-1β (IL-1β), interleukin-6 (IL-6) and tumor necrosis factor (TNF)) in pain-related brain regions: namely the periaqueductal gray (PAG) [23, 24], hypothalamus [24], hippocampus [25–29], prefrontal cortex [23, 27, 30, 31], nucleus accumbens (NAcc) [32] and rostral ventromedial medulla [33]. Furthermore, some of these studies have demonstrated that nerve injury-induced pro-inflammatory cytokine release in the brain is responsible for behavioural changes, including memory deficits [29, 34], reduced conditioned place-preference to morphine [32], and depressive-like behaviour [23].

Work from our laboratory over a number of years has shown consistently that sciatic nerve chronic constriction injury (CCI), a commonly used model of neuropathic pain, triggers persistent changes in resident-intruder social interactions in only a subgroup of rats (~30 %), termed *Pain and disability* [35–40]. This subgroup of rats also exhibits changes in sleep-wake cycle regulation and neuro-endocrine disruptions in both HPA and hypothalamo-pituitary-thyroid axes [37, 41, 42]. Furthermore, we have shown recently that a specific neuro-immune signature of gene expression in the spinal cord characterises these *Pain and disability* rats at both day two and day six following CCI [43]. Despite only a subgroup of rats showing disabilities, all CCI rats show equal levels of thermal and mechanical allodynia and hyperalgesia [1, 37]. Simply put, sensory changes are decoupled from the expression of altered social interactions, sleep-wake cycle disruption and neuro-endocrine dysfunction.

The emerging appreciation of the importance of the neuro-immune interface in: (i) regulating affective-motivational and cognitive function and; (ii) the expression of the neuropathic pain state, raises the important question of whether individual differences in the expression of disabilities following nerve injury, as seen in the *Pain and disability* rats, reflects distinct neuro-immune signatures which can be detected in both the periphery and the spinal cord. The experiments reported here are directed at answering this question.

Outbred rats underwent CCI or sham surgery and were characterised using the resident-intruder social interactions paradigm for injury-induced disabilities prior to the following: (i) structural quantification of the degree of nerve damage following CCI by examining myelin thickness, immunoreactivity of S100 (a Schwann cell marker) and assessing the number of dorsal root ganglia (DRG) neurons expressing the stress transcription factor, activating transcription factor 3 (ATF3); (ii) quantification of the number of both innate (macrophages) and adaptive immune cells (T lymphocytes) present in the sciatic nerve and DRG; and (iii) quantification of the protein levels of several cytokines in the ipsilateral sciatic nerve, ipsilateral DRG and the L4-L5 spinal cord segments. We found that *Pain and disability* rats had a distinct immune response at both peripheral and spinal cord sites compared to rats without disabilities, despite equal levels of damage to the sciatic nerve and degree of allodynia.

## Methods

### Animals

All experimental procedures were carried out in accordance with the guidelines of the NHMRC “Code for the care and use of animals in research in Australia” and the “Ethical guidelines for investigations of experimental pain in conscious animals” laid down by the “International Association for the Study of Pain” [44]. Furthermore, the “University of Sydney animal care and ethics committee” (AEC) approved all procedures (#AEC numbers 3176, 3920 and 4852). We also followed the ARRIVE guidelines for “Animal Research: Reporting In Vivo Experiments” (http://www.nc3rs.org.uk/arrive-guidelines). All procedures were designed to minimise the intensity and duration of animal suffering as well as animal numbers, within the context of addressing the experimental aims.

Experiments were performed on 82 outbred, male Sprague–Dawley rats, (ARC, Australia) weighing 220–320 g on the day of CCI. Rats were housed individually in clear Perspex cages in an animal house maintained on a reversed 12/12 h light/dark cycle (lights on at 1900 h) with food and water available *ad libitum*. Behavioural analyses were conducted during the dark phase of the circadian cycle. Room temperature was maintained at 22 (±1) °C.

### “Resident-intruder” social interactions testing

The resident-intruder paradigm used in these studies consisted of habituating the “resident” to its home-cage for one week, before the introduction of an unfamiliar sex, age and weight-matched “intruder” rat (see [37] for complete details). Rats were randomly assigned as residents or intruders. Social interactions between residents and intruders were analysed for six days before CCI, followed by a further six days of testing after CCI (*n* = 62) or sham surgery (*n* = 16). On the day of CCI, no behavioural testing was conducted. Social interactions were video recorded for 6 min at approximately the same time during the dark phase each day. Resident rats never encountered the same intruder more than twice and never on consecutive days throughout testing.

### Chronic constriction injury of the sciatic nerve

Sciatic nerve CCI was performed in a manner identical to that first described by Bennett and Xie [45]. Briefly, anaesthesia was induced with 5 % halothane/isoflurane in 100 % O_2_ (Lyppard, Castle Hill, NSW, Australia) and maintained during surgery via a custom made facemask (2 % in 100 % O_2_). The right sciatic nerve was exposed by blunt dissection through the biceps femoris and four ligatures (chromic gut, 5.0, Johnson & Johnson Medical, North Ryde, NSW, Australia) were loosely tied, 1 mm apart, just proximal to the trifurcation of the sciatic nerve. Constriction was minimal to cause “visible retardation, but not arrest, of the epineural vasculature” as originally defined [45]. Sham rats had the sciatic nerve exposed but not ligated. The incision was sutured (Mersilk, 5.0, Johnson & Johnson Medical, North Ryde, NSW, Australia) and iodine solution (Povidone-Iodine, Orion Laboratories, Balcatta, WA, Australia) and triple antibiotic powder (Tricin, Sigma-Aldrich, Castle Hill, NSW, Australia) were applied topically. Each rat was observed closely during its recovery period and during the 24 h following its return to the home-cage.

### Behavioural analyses

Resident behaviour during the 6 min test period was quantified within four mutually exclusive categories. *Dominance behaviour*: standing on top of the supine intruder, back or lateral attack with biting targeted at the neck or back of the intruder and chasing the intruder. *Social behaviour*: investigation or sniffing of intruder with particular focus on anogenital region. *Non-social behaviour*: cage exploration and self-grooming. *Submissive behaviour*: defensive alerting/freezing and defensive sideway or supine posture upon the approach of the intruder. These categories were identical to those used previously [37].

Development of stable resident-intruder interactions requires prior intruder exposure. Therefore, the behaviour of each resident on the post-CCI days was compared with its behaviour on the three days immediately prior to CCI (i.e. pre-CCI days 4–6) once stable interactions had been established. Resident rats were then categorised into three subgroups, based upon changes in the duration of dominance behaviour post-CCI, in keeping with previous studies: (i) *Pain alone* (*n* = 24): no change in the duration of dominance behaviour post-CCI, compared to pre-CCI; (ii) *Pain and disability* (*n* = 19): a reduction of at least 30 % in the duration of dominance behaviour on at least 5 out of 6 post-CCI days, compared to pre-CCI days; and (iii) *Pain and transient disability* (*n* = 19): an initial transient reduction of at least 30 % in the duration of dominance behaviour on the first 3–4 days post-CCI, compared to pre-CCI, followed by a return to pre-CCI levels (days 4/5–6).

### Mechanical withdrawal threshold testing

Testing was conducted in the dark phase under red light, at least 1 h after resident-intruder interactions. Rats were habituated to the behavioural testing apparatus for at least 30 min before data collection. Three baseline pain behaviour measurements were made prior to nerve injury, as well as two post-injury time-points (post-CCI day 2/3 or 4/5). Sensitivity to mechanical stimuli was assessed using a dynamic plantar von Frey anesthesiometer (Ugo Basile, Varese, Italy). The von Frey filament was applied to the mid-plantar surface, and the mechanical withdrawal threshold (in grams) of each hind-paw was calculated as the mean of five trials. The interval between trials on the same paw was at least 5 min.

### Immunohistochemistry

Immediately following resident-intruder testing on day 6, 14-19 rats per behavioural group and 11 shams were deeply anaesthetised with sodium pentobarbitone (i.p., Lethabarb, 120 mg/kg, Lyppard) and perfused transcardially with heparinised 0.9 % saline, followed by 4 % paraformaldehyde in acetate-borate buffer (pH 9.6; 4 °C). The sciatic nerve and L4 DRG were removed and post-fixed for 1 h before being cryoprotected in 30 % sucrose in PBS (pH 7.4), with 0.05 % sodium azide, and stored at 4 °C. Tissues were cryosectioned, with the sciatic nerve cut longitudinally (14 μm) and the DRG cut coronally (10 μm). Ten series of sections were collected onto slides at intervals of 1 in 10, with slides stored at −20 °C until use. Staining was performed directly onto the slides, with the sections first washed in 100 % ethanol for 10 min. Sections were then twice rinsed in distilled water, before one wash in PBS. For T cell receptor αβ (TCRαβ) staining, an additional 3 min incubation with acetone was followed by three PBS washes. The sections were blocked for 30 min in PBS containing 0.05 % Tween-20 and 5 % normal horse serum (NHS) (Sigma-Aldrich, Castle Hill, NSW, Australia). Sciatic nerve and DRG sections were stained for T lymphocytes with mouse-anti-rat TCRαβ (1:250, clone R73, BD Bioscience, North Ryde, NSW, Australia) and for macrophages with mouse-anti-rat CD68 (1:250, clone ED1, Serotec, distributed by Abacus ALS, Meadowbrook, QLD, Australia) in PBS containing 5 % bovine serum albumin (BSA) and 0.05 % Tween-20 for 2 h at room temperature. Additionally, sciatic nerves were stained with rabbit-anti-rat-S100 (1:500, Sigma-Aldrich, Castle Hill, NSW, Australia), a Schwann cell structural protein, also in PBS containing 5 % BSA and 0.05 % Tween-20 for 2 h at room temperature. The DRG were stained with rabbit-anti-rat-ATF3 (1:500, Santa Cruz Biotechnology, Santa Cruz, CA, USA) in 5 % NHS with 0.15 % Triton-X for 16 h. Following primary incubations, sections were rinsed three times in PBS and then incubated with Alexa488 conjugated donkey anti-mouse (1:100, Jackson, West Grove, PA, USA) or Cy3 conjugated donkey anti-rabbit (1:500, Jackson, West Grove, PA, USA) for 1 h in the same buffer as the primary antibody. The sections were then washed three times in PBS before being cover-slipped with fluorescent mounting medium with DAPI (Dako, North Sydney, NSW, Australia).

#### Image analysis

An investigator blinded to the behavioural group of each rat undertook all image analyses. Sections were viewed on a fluorescence microscope (BX51, Olympus, Tokyo, Japan) and images captured using a digital camera (DP70, Olympus, Tokyo, Japan). Multiple images were taken from random fields of view, on each of four or five sections from each rat. The images were non-overlapping and entirely within the boundary of the nerve or in areas of the DRG containing >90 % cell bodies.

TCRαβ and ED1 immunoreactivity (IR) in injured sciatic nerves was quantified from 12 images captured at the site of injury (i.e. the fields of view in the 1 mm between two chromic gut sutures and within 1 mm of the most proximal and distal sutures). Images from an identical anatomical position, with respect to the sciatic nerve trifurcation, were also captured from the sham rats.

Immune cells (i.e. TCRαβ + T lymphocytes in sciatic nerves and DRG and ED1-IR macrophages in the DRG) or neuronal nuclei (i.e. ATF3-IR in the DRG) were counted manually using the ImageJ cell counter plug-in (NIH, Bethesda, MD, USA). TCRαβ + and ED1+ cell counts were given as cells per mm^2^, adjusted from total area sampled from 12 images, and the size of the field of view using a × 40 objective lens. ATF3-IR neuronal nuclei were expressed as a percentage of total neurons counted across ten images, thus normalising for different numbers of neurons counted. However, a minimum number of 300 neurons were assessed for each rat.

Where cell numbers were numerous (i.e. ED1 in the nerve) or individual cells overlapped and continuous cell borders could not be distinguished (i.e. S100 in the nerve), densitometry analysis was performed using ImageJ. A normalisation process was used for densitometry measurements. The brightness and contrast setting in ImageJ were set to standardise the levels of the background and the image was converted to a 16-bit black-and-white image, so that the auto-threshold function could be applied to the images prior to measuring the %IR area. For ED1, the mean densitometry measurements were calculated from 12 images per rat. For S100-IR, the reduction in staining at the injury site was assessed by comparing eight images captured within 1 mm of the injury site to eight images captured 4 mm proximal to the injury site where no damage was evident. Thus, the mean %IR at the site of injury was divided by the %IR at the uninjured proximal site to calculate S100-IR ratio. In the sham rats, images of S100 were taken at identical anatomical positions within the nerve in order to create a comparison S100-IR ratio.

### Electron microscopy

Five rats from both *Pain alone* and *Pain and disability* groups, as well as four control nerves from uninjured rats were used for electron microscopy. Immediately following resident-intruder testing on day six, rats were deeply anaesthetised with sodium pentobarbitone (i.p., Lethabarb, 120 mg/kg) and perfused transcardially with heparinised 0.9 % saline. A 2.0-cm span of sciatic nerve was freshly dissected from each rat and placed in Karnovsky’s fixative solution (2.5 % glutaraldehyde, 2 % paraformaldehyde in 0.1 M phosphate buffer, pH 7.4) for 1 h. Nerves were post-fixed in 2 % osmium tetroxide for 30 min. Following fixation, a section of each sciatic nerve was cut which was exactly 1 mm proximal to the most proximal ligature. Nerve sections were dehydrated in an ascending series of alcohol before embedding. Nerves were placed in a 1:1 mixture of Spurr’s resin and 100 % ethanol and incubated for 1 h on a rotating platform. The solution was replaced with 100 % Spurr’s resin and incubated on the rotator overnight. The nerves were transferred to small tubes to act as moulds, which were filled up with Spurr’s resin and transferred to an oven at 65 °C to polymerise overnight. Embedded nerves were sectioned at 70 nm on an ultra-microtome from a position 1 mm proximal to the injury site before being mounted onto support grids for imaging.

For each rat, ten randomly selected non-overlapping fields of view were captured at × 1200 magnification using a transmission electron microscope (JEM-1011, Jeol, Tokyo, Japan) and digital camera (Orius 833, Gatan, Pittsburgh, PA, USA). A second investigator blinded to the behavioural groups of the rats undertook manual analysis of images. Measurements of myelin thickness from 100 axons across the ten images were taken, and a mean thickness was calculated. Axons of all classes (Aα, Aβ and Aδ) were selected for measurement if they intersected with an equatorial line. The total number of axons per 70 μm^2^ field of view was counted and a mean calculated across the ten images for an individual rat.

### Multiplex cytokine assays

#### Tissue preparation

On post injury day seven, 4–5 rats per group were deeply anaesthetised with sodium pentobarbitone (i.p., Lethabarb, 120 mg/kg) and perfused with heparinised 0.9 % saline. The injured sciatic nerve, its L4 and L5 DRG, and segments L4-L5 of the spinal cord (ipsilateral and contralateral to the injury) were dissected, snap frozen in liquid nitrogen, and then stored at −80 °C.

Tissue was homogenised using a total protein extraction kit (Merck Millipore, Bayswater, VIC, Australia) consisting of 1× protease inhibitors in TM buffer (HEPES, MgCl_2_, KCl, EDTA, sucrose, glycerol, sodium deoxycholate, NP-40, sodium orthovanadate). TM buffer, 2.5 ml per gram of tissue, was added in two batches, each followed by 5 min incubation on ice. Homogenising beads (5 mm, stainless steel; Qiagen, Melbourne, VIC, Australia) were used to mechanically disrupt samples using a TissueLyser LT (Qiagen, Hilden, Germany) at 50 Hz for 20 s, repeated 6–8 times until fully homogenised. Tissue homogenates were mixed at 4 °C for 20 min before centrifugation at 11,000 rpm at 4 °C for 20 min. The supernatant was isolated from the pellet before a second centrifugation to obtain a completely clear supernatant. The supernatant was assayed for total protein concentration using an EZQ Protein Quantification kit (Invitrogen, Mount Waverley, VIC, Australia) according to the manufacturer’s instructions. Protein samples were stored at −20 °C until cytokine assay.

#### Running the multiplex cytokine assays

Custom-made multiplex cytokine assays (Bio-Plex Pro Rat 8-plex, Bio-Rad Laboratories, Gladesville, NSW, Australia) were used, according to the manufacturer’s instructions, to determine the concentration of IL-1β, IL-6, IL-10, IL-17A, IL-18, TNF, IFN-γ and MCP-1 in each of the homogenates collected. Briefly, tissue homogenates were thawed on ice and standardised to 1600 μg/mL, diluting in TM buffer. Samples were mixed 1:2 with sample diluent to bring the concentration to 800 μg/mL, and kept at 4°C until used. To start the assay, 50 μl of vortexed magnetic microbeads were added to each well. Beads were then washed twice with Bio-Plex Pro wash buffer using a magnetic plate washer (Tecan HydroFlex, Crailsheim, Germany). Fifty microliters of standards and samples were vortexed and added to the wells. Plates were then sealed and kept in the dark, before being incubated at room temperature for 1 h with agitation on a plate mixer. Plates were washed three times and 25 μl of vortexed detection antibodies were added to each well prior to 30 min incubation. Plates were washed three times and 60 μl of vortexed streptavidin-phycoerythrin reporter (SA-PE) was added to each well, before incubation for 10 min. Next, plates were washed three times before adding 125 μl assay buffer to each well. Plates were then sealed and stored at 4 °C until acquisition. Data were collected using a Bio-Plex 100 suspension array system (Bio-Rad Laboratories, Gladesville, NSW, Australia). Calibration kits were run before reading each plate. Plates were agitated at 1100 rpm for 30 s prior to reading. Standard curves were optimised and sample cytokine concentrations determined using Bio-Plex Manager software (v6.0 Bio-Rad Laboratories, Gladesville, NSW, Australia).

### Statistical analysis

All data are presented as group means (±SEM). The effects of time and post-injury behavioural group on dominance and non-social behaviours were analysed using a two-way ANOVA with Bonferroni *post hoc* comparisons, between each of the behavioural groups and shams. For all electron microscope and immunohistological measurements, an omnibus one-way ANOVA with Bonferroni *post hoc* comparisons was used to compare all groups. For cytokine expression levels, sham rats were compared to the CCI rats using a one-way ANOVA. A second test on the cytokine expression levels used an omnibus one-way ANOVA with Bonferroni *post hoc* comparisons between each behavioural subgroup and shams. The data from individual rats were also analysed using linear regression to determine significant relationships between changes in dominance and changes in mechanical withdrawal threshold with: (i) ATF3-IR cells; (ii) TCRαβ-IR; (iii) ED1-IR; and (vi) cytokine expression levels, in each of the neural tissues collected. Only where statistically significant correlations occurred are the data shown (i.e. Fig. 5f). All other correlations were not statistically significant. For all statistical analyses, family-wise error rate was corrected using the Benjamini-Hochberg procedure, with a false discovery rate of *q* <0.05 to identify significance. A “family of analyses” was considered to be where the same biological material was used for a group of independent analyses (i.e. analyses of multiplex cytokine data conducted using the same tissue sample or analyses of multiple immunohistochemistry analyses conducted on the same nerve or DRG).

**Fig. 1:**
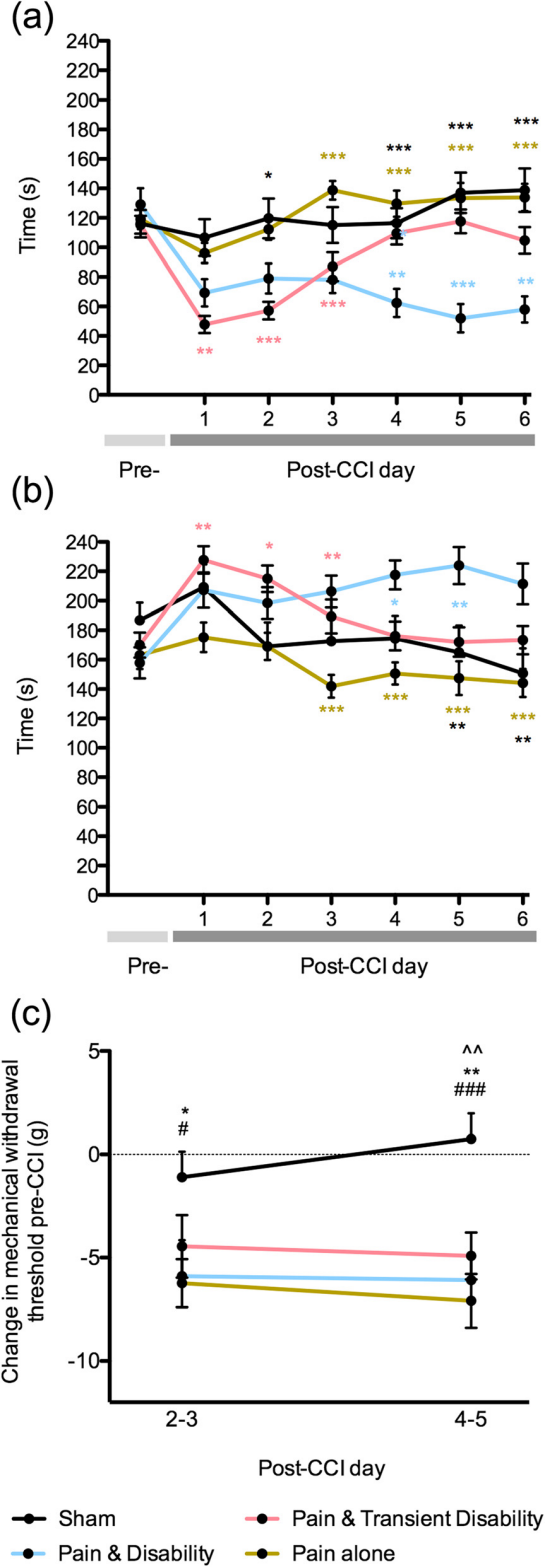
The duration of **a** dominance and **b** non-social behaviours during daily resident-intruder social interactions in sham controls or rats classified as having *Pain alone*, *Pain and disability* and *Pain and transient disability* six days after CCI (*n* = 16–24 per group). **c** The reduction in mechanical withdrawal threshold in the injured hind-paw, compared to the pre-CCI baseline, at two time-points (days 2–3 and days 4–5) after CCI. Statistically significant differences in social interactions are indicated by ^*^ *P* <0.05, ^**^ *P* <0.01 and ^***^ *P* <0.001, with *black asterisk* indicating differences between sham vs. *Pain and disability*, *brown asterisk Pain alone* vs. *Pain and disability*, *pink asterisk* indicating *Pain alone* vs. *Pain and transient disability* and *blue asterisk* indicating *Pain and disability* vs. *Pain and transient disability*. Significant differences in withdrawal threshold compared to shams are indicated by *P <0.05 and **P <0.01 for *Pain and disability*, ^#^ *P* <0.05 and ^##^ *P* <0.01 for *Pain alone*, and ^^^^ *P* <0.01 for *Pain and transient disability*

## Results

### CCI triggers three distinct patterns of altered social interactions

Consistent with our previous findings, three distinct patterns of behaviour emerged following CCI. Twenty-four residents were classified in the *Pain alone* group, maintaining dominance behaviours towards the intruder after CCI. Nineteen of the residents were classified in the *Pain and disability* group, that is, they displayed a significant reduction (>30 %) in the duration of dominance behaviours on at least 5 post-CCI days. The remaining 19 residents were classified in the *Pain and transient disability* group, displaying a transient and significant reduction (~30 %) in the duration of dominance on days 1–3 post-CCI, after which dominance behaviour returned to pre-injury levels on days 4–6 after CCI.

Figure 1a, b shows the trajectories of dominance and non-social behaviours, respectively, in each behavioural group following CCI. There was a significant reduction in dominance behaviour in the *Pain and disability* rats compared to *Pain alone* rats on days 3–6 post-CCI (*P* <0.001), which was mirrored by a significant increase in non-social behaviour (*P* <0.001). On post-CCI days 1–3, the *Pain and transient disability* group had a significant reduction in dominance behaviour (*P* <0.01–0.001) and an increase in non-social behaviour (*P* <0.05–0.01) compared to *Pain alone*, although their behaviour returned to pre-injury levels by days five and six post-CCI.

**Fig. 2:**
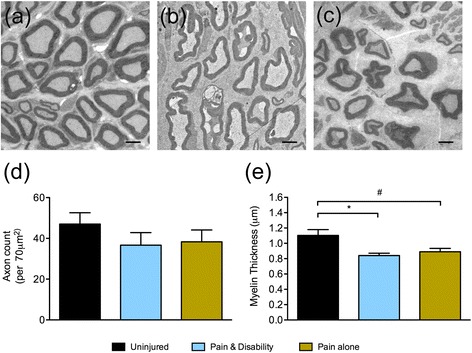
Representative electron micrographs of axons located 1 mm proximal to the injury site six days after CCI from **a** uninjured, **b** *Pain and disability* and **c** *Pain alone* rats. Scale bars represent 2 μm. **d** The mean axon count per 70 μm^2^ field of view, and **e** The mean myelin thickness of 100 axons from uninjured controls (*n* = 4), as well as from *Pain alone* and *Pain and disability* rats (*n* = 5 per group). Significant differences compared to uninjured controls are indicated by *P <0.05 for *Pain and disability* and ^#^ *P* <0.05 for *Pain alone*

Figure 1c shows the mean reduction in mechanical withdrawal thresholds in the injured hind-paw at two time-points post-CCI. In keeping with our previous observations, all behavioural groups show a significant reduction in paw withdrawal threshold compared to shams. Thus, the expression of behavioural disability does not relate to the degree of mechanical allodynia induced by CCI.

### CCI-induced structural changes are equal in all CCI disability groups

Ultrastructural changes in the injured nerve were assessed using transmission electron microscopy, 1 mm proximal to the site of sciatic nerve ligation, in rats with or without behavioural disability on day six post-CCI. Uninjured nerves have tightly packed axons of a uniform cross-sectional morphology (Fig. 2a). In contrast, injured nerves have visible oedematous spaces between axons, as well as axons with abnormal ruffled and blebbed morphology (Fig. 2b, c). Although after CCI the number of axons does not significantly decrease (Fig. 2d), myelin thickness is significantly reduced in both *Pain alone* and *Pain and disability* rats (Fig. 2e, *P* <0.05). Therefore, the extent of structural change in axons located proximal to the injury site did not differ between the two disability groups assessed.

**Fig. 3:**
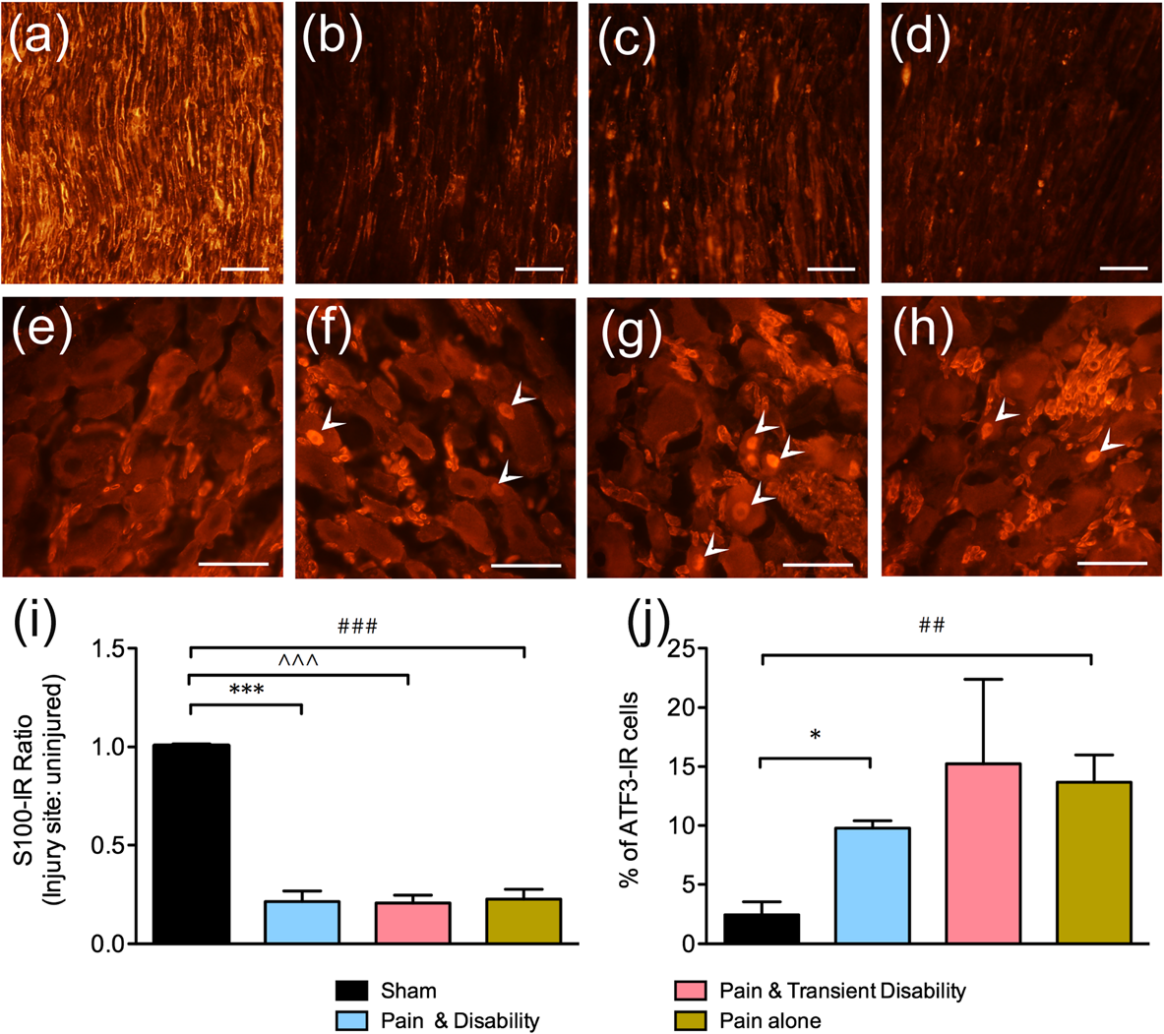
Representative immunoreactivity (IR) of the Schwann cell marker, S100, at the site of sciatic nerve injury in **a** sham, **b** *Pain and disability,* **c** *Pain and transient disability* and **d** *Pain alone* rats on day six after CCI. Scale bars represent 50 μm. Representative ATF3-IR, a marker of cellular stress, in the ipsilateral L4 DRG neurons in **e** sham, **f** *Pain and disability*, **g** *Pain and transient disability* and **h** *Pain alone* rats on day six after CCI. Arrowheads indicate ATF-IR nuclei. Scale bars represent 50 μm. **i** The ratio of S100-IR at the site of injury compared to an uninjured site following CCI (*n* = 11–14 per group). **j** The percentage of neurons with ATF3-IR nuclei compared to the total neurons counted in the ipsilateral L4 DRG after CCI (*n* = 4–7 per group). Significant differences compared to sham controls are indicated by *P <0.05 and ***P <0.001 for *Pain and disability,* ^##^ *P* <0.01 and ^###^ *P* <0.001 for *Pain alone*, and ^^^^^ *P* <0.001 for *Pain and transient disability*. All significant differences have been corrected for family-wise error rate with the Benjamini-Hochberg procedure, with a false discovery rate of *q* <0.05

Using S100, a marker for Schwann cells, the extent of demyelination at the site of ligation was investigated six days after CCI. In sham rats, intense S100-IR shows Schwann cells encapsulating axons, appearing as hollow tubes, arranged closely in a uniform direction (Fig. 3a). In all CCI rats at the site of ligation, morphological differences in Schwann cells were detected; the cells showed a granular appearance and disorderly arrangement, accompanied by a large reduction in S100-IR (Fig. 3b–d). The degree of loss of Schwann cells was quantified by comparing S100-IR at the site of nerve injury to an uninjured site located more proximally, thus creating an S100-IR ratio. In sham rats, this ratio was approximately 1, as the nerves displayed intense S100 along their length. However, in CCI rats of all behavioural groups, this ratio was less than 1 and was significantly different to the shams (Fig. 3i, *P* <0.001). Thus, the extent of the loss of Schwann cells and resulting demyelination appears similar in rats from each of the three disability groups.

**Fig. 4:**
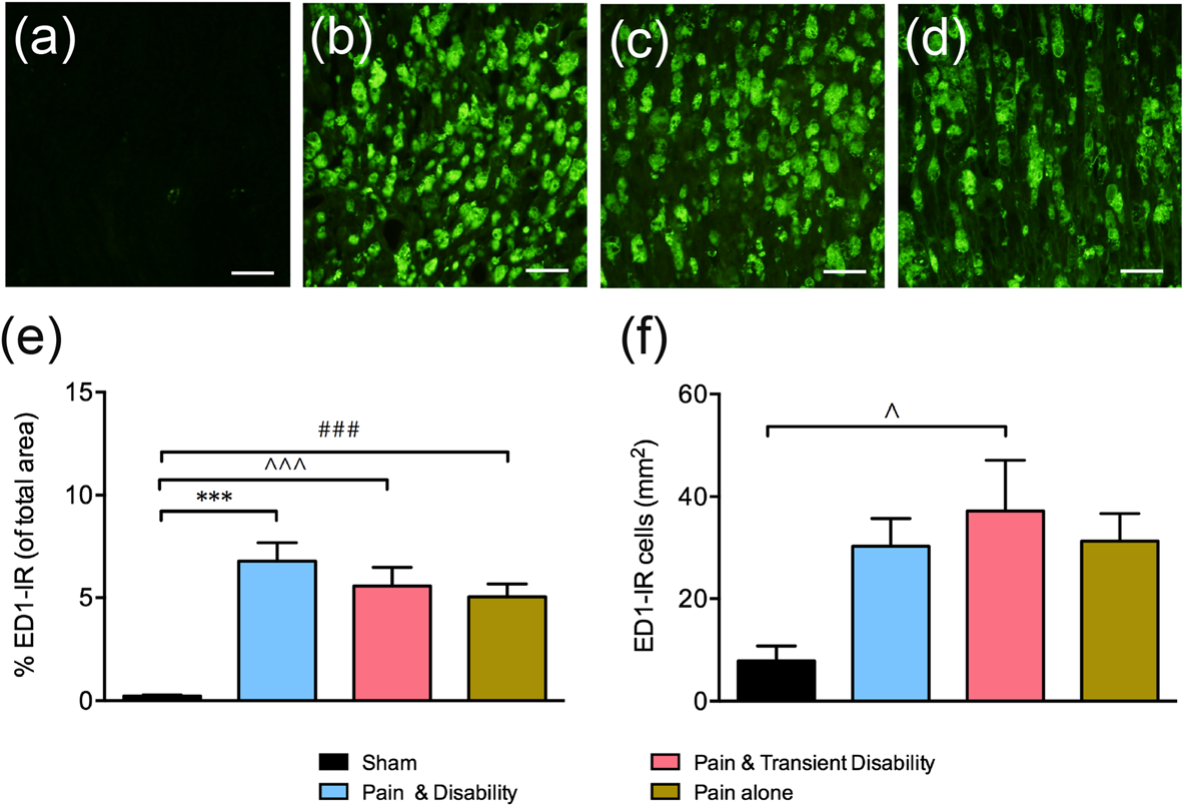
Representative ED1-IR, a macrophage marker, at the site of sciatic nerve injury in **a** sham, **b** *Pain and disability*, **c** *Pain and transient disability* and **d** *Pain alone* rats on day six after CCI. Scale bars represent 20 μm. **e** The percentage of ED1-IR (of total area) at the site of nerve injury after CCI (*n* = 9–13 per group). Scale bars represent 20 μm. **f** The number of ED1-IR cells per mm^2^ in the ipsilateral L4 DRG after CCI (*n* = 4–8 per group). Significant differences compared to sham controls are indicated by **P <0.01 for *Pain and disability,* ^#^ *P* <0.05 for *Pain alone*, and ^^^ *P* <0.05 and ^^^^ *P* <0.01 for *Pain and transient disability*. All significant differences have been corrected for family-wise error rate with the Benjamini-Hochberg procedure, with a false discovery rate of *q* <0.05

Using the transcription factor ATF3, a marker of cellular stress, damage to individual neurons in the DRG was investigated six days after CCI. In sham rats there were very few DRG neurons that contained ATF3-IR nuclei (Fig. 3e). In contrast, many ATF3-IR nuclei were seen in the DRG of rats after CCI (Fig. 3f–h). The percentage of ATF-IR cells was significantly increased in rats with *Pain and disability* (*P* <0.05) and *Pain alone* (*P* <0.01) compared to shams. Due to high variance in the *Pain and transient disability* group, there was no significant difference compared to shams, although the mean of 15.2 ± 6.4 % was greater than the other CCI groups (*Pain alone*, 13.7 ± 2.1 % and *Pain and disability*, 9.8 ± 0.55 %) and shams (2.3 ± 0.95 %) (Fig. 3j). There was no relationship between the numbers of ATF3-IR cells and either reduction in dominance behaviour or withdrawal thresholds after CCI; it is unlikely therefore to be directly related to the expression of either allodynia or disability.

### CCI induces a robust immune response in the peripheral nervous system, which is greatest in *Pain and disability* rats

Using the macrophage marker ED1, the number of cells infiltrating the injured sciatic nerve and its L4 DRG were assessed six days after CCI. In the nerves of sham rats, there was almost no ED1-IR (Fig. 4a). At the site of sciatic nerve injury dense ED1-IR was seen, indicative of surface staining of infiltrating macrophages (Fig. 4b–d). After CCI, quantification of the percentage ED1-IR at the injury site revealed significant increases in rats with *Pain and disability* (6.79 ± 0.9 %, *P* <0.001), *Pain alone* (5.0 ± 0.6 %, *P* <0.001) and *Pain and transient disability* (5.6 ± 0.9 %, *P* <0.001) compared to shams (0.23 ± 0.04 %, Fig. 4e). The number of ED1-IR cells in the L4 DRG of CCI rats (33 ± 3.9 cells per mm^2^) was greater than in sham rats (11 ± 3.0 per mm^2^). However, this difference reached significance in only the *Pain and transient disability* rats (*P* <0.05) (Fig. 4f). Thus, a large number of macrophages infiltrate both the sciatic nerve and its L4 DRG after CCI, with the highest number in the *Pain and disability* rats (sciatic nerve), and *Pain and transient disability* rats (DRG).

**Fig. 5:**
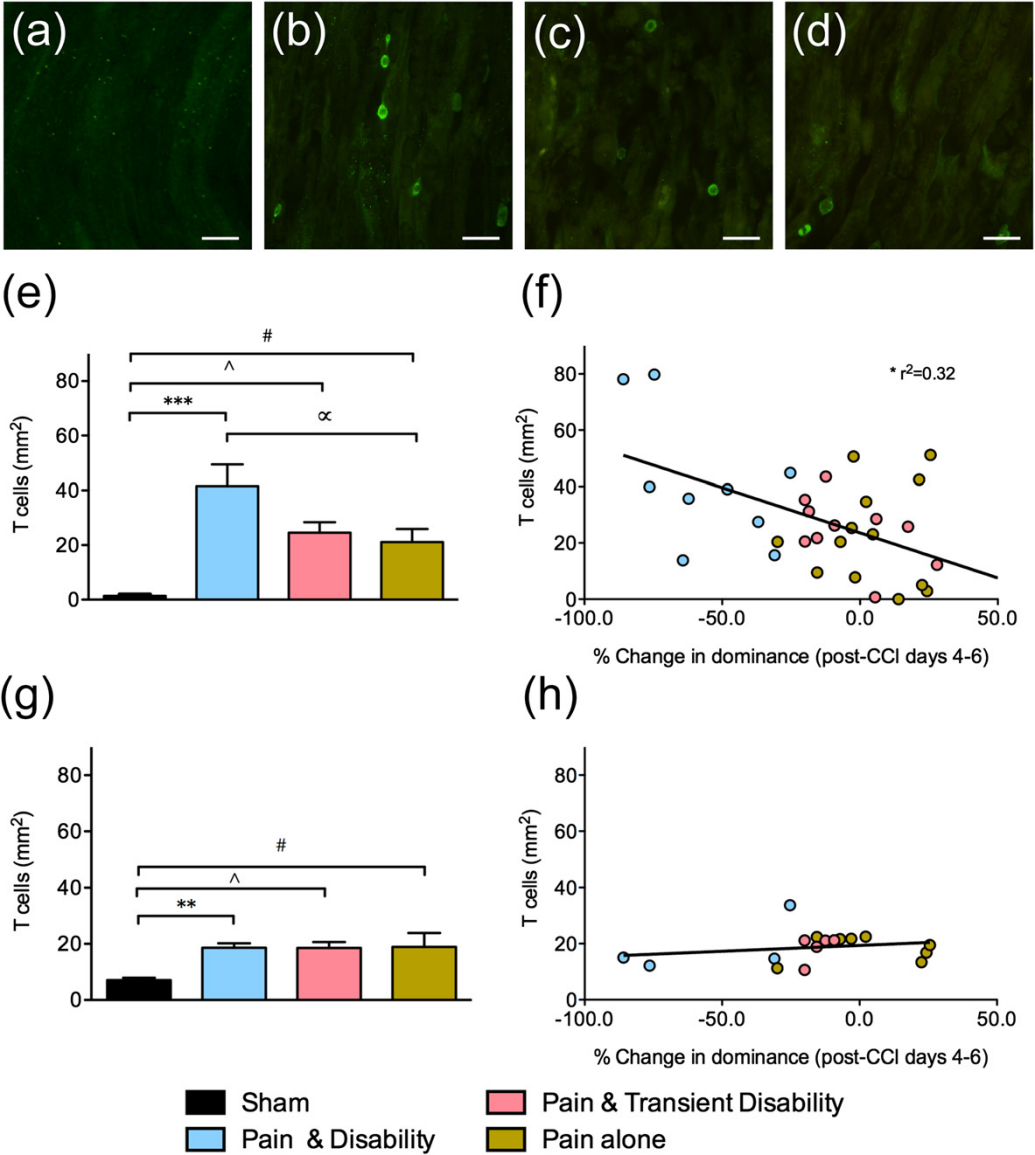
Representative TCRαβ-IR, a T lymphocyte marker, at the site of sciatic nerve injury in **a** sham, **b** *Pain and disability*, **c** *Pain and transient disability* and **d** *Pain alone* rats on day six after CCI. Scale bars represent 20 μm. **e** The number of T lymphocytes per mm^2^ at the site of nerve injury after CCI (*n* = 9–14 per group). **f** The relationship between T lymphocytes in the sciatic nerve and the percentage change in dominance (from pre-CCI days 4–6 to post-CCI days 4–6) after CCI, in 33 rats. **g** The number of T lymphocytes per mm^2^ in the ipsilateral L4 DRG after CCI (*n* = 4–8 per group). **h** The relationship between the number of T lymphocytes in the ipsilateral L4 DRG and the percentage change in dominance (from pre-CCI days 4–6 to post-CCI days 4–6) after CCI, in 17 rats. Significant differences compared to sham controls are indicated by **P <0.01 and ***P <0.001 for *Pain and disability,* ^#^ *P* <0.05 for *Pain alone* and ^^^ *P* <0.05 for *Pain and transient disability*. Significant difference between *Pain and disability* and *Pain alone* is indicated by ∝*P* <0.05. All significant differences have been corrected for family-wise error rate with the Benjamini-Hochberg procedure, with a false discovery rate of *q* <0.05

Using T cell receptor αβ-IR (TCRαβ-IR), the number of T lymphocytes infiltrating the injured sciatic nerve and DRG were assessed six days after CCI. In the nerves of sham rats, there was almost no TCRαβ-IR (Fig. 5a). However, there was significant TCRαβ-IR visible on the surface of cells at the site of sciatic nerve injury, indicative of infiltrating T lymphocytes (Fig. 5b–d). Counts of individual cells expressing TCRαβ within the injury site revealed significant increases in rats with *Pain and disability* (41.6 ± 7.4 cells/mm^2^, *P* <0.001), *Pain alone* (21.1 ± 4.6 cells/mm^2^, *P* <0.05) and *Pain and transient disability* (24.6 ± 3.6 cells/mm^2^, *P* <0.05) compared to shams (1.4 ± 0.6 cells/mm^2^, Fig. 5e). However, there were also significantly more T lymphocytes in *Pain and disability* rats compared to *Pain alone* rats (*P* <0.01). A linear regression analysis revealed a significant positive relationship between the number of T lymphocytes at the injury site and the percentage change in dominance after CCI (Fig. 5f, *r*^2^ = 0.32). After CCI, T lymphocyte counts were significantly increased in the L4 DRG of rats with *Pain and disability* (*P* <0.01), *Pain alone* (*P* <0.05) and *Pain and transient disability* (*P* <0.05) (Fig. 5g). There was no significant relationship between the number of T lymphocytes in the L4 DRG and the percentage change in dominance after CCI (Fig. 5h). Thus, the number of T lymphocytes infiltrating the injured nerve, but not the DRG, in response to CCI is positively correlated with the expression of disability.

The protein expression of a panel of six pro-inflammatory cytokines (IL-1β, IL-6, IL-17A, IL-18, TNF, IFN-γ), the chemokine, MCP-1 and the anti-inflammatory cytokine, IL-10, were assessed using a multiplex assay in the sciatic nerve seven days after CCI (Table 1). CCI triggered a massive increase in IL-1β, which exceeded the sensitivity of the assay. Further, the levels of IFN-γ were below the threshold for detection in sham-injured rats but were reliably detected in all behavioural groups following CCI. IL-6 and MCP-1 were significantly increased in CCI rats compared to sham rats (*P* <0.05 and *P* <0.001, respectively). Conversely, IL-17A was significantly decreased in CCI compared to sham (*P* <0.001). Looking at the disability subgroups, the *Pain and disability* group was the only group to have a significant increase in IL-6 (151.8 ± 33.9 pg/ml) compared to sham rats (19.1 ± 3.4 pg/ml, *P* <0.01). Moreover, the concentration of IL-6 in this group was approximately two-fold greater than rats with *Pain alone* (82.5 ± 20.2 pg/ml) and *Pain and transient disability* (61.4 ± 13.1 pg/ml). MCP-1 expression was increased in only the *Pain and disability* group (546.8 ± 55.2 pg/ml) compared to shams (151.2 ± 42.7 pg/ml, *P* <0.001). CCI triggered a select decrease in sciatic nerve IL-17A in rats without persistent disability, that is, *Pain and transient disability* and *Pain alone* rats compared to sham (*P* < 0.01 and *P* <0.05, respectively). These data indicate a substantial pro-inflammatory cytokine response in the sciatic nerve seven days after CCI, with *Pain and disability* rats showing an increase in IL-6 and MCP-1, and *Pain and transient disability* and *Pain alone* rats showing a decrease in IL-17A.

**Table 1:** Mean cytokine concentration (pg/mL) (±SEM) in the ipsilateral sciatic nerve seven days following CCI

Cytokine	Sham (*n* = 5)	Combined CCI (*n* = 14)	*Pain and disability* (*n* = 5)	*Pain and transient disability* (*n* = 4)	*Pain alone* (*n* = 5)
IL-1β	73.4 ± 8.2	>3860	>3860	>3860	>3860
IL-6	19.1 ± 3.4	101.2 ± 16.6*	151.8 ± 33.9**	61.4 ± 13.1	82.5 ± 20.2
IL-10	189.2 ± 36.2	162.4 ± 16.1	135.6 ± 19.4	157.0 ± 26.0	168.7 ± 10.8
IL-17A	55.7 ± 1.6	35.2 ± 2.4***	37.9 ± 3.4	30.3 ± 5.0**	36.4 ± 4.4*
IL-18	77.8 ± 7.1	71.1 ± 2.6	73.2 ± 6.1	67.6 ± 3.5	71.6 ± 4.1
TNF	19.5 ± 1.2	77.6 ± 12.1*	96.0 ± 26.3	61.2 ± 8.3	72.4 ± 23.4
IFN-γ	<5	33.5 ± 3.8	35.9 ± 9.1	34.0 ± 0.6	31.3 ± 5.4
MCP-1	151.2 ± 42.7	440.2 ± 34.1***	546.8 ± 55.2***	367.9 ± 45.3	391.5 ± 51.5

Statistically significant differences compared to sham are indicated by *P <0.05, **P <0.01 and ***P <0.001. All significant differences have been corrected for family-wise error rate with the Benjamini-Hochberg procedure, with a false discovery rate of *q* <0.05

The protein expression of the same panel of cytokines was assessed in the L4 and L5 DRG ipsilateral to the injury seven days after CCI (Table 2). IL-6 and MCP-1 were significantly increased in CCI rats compared to sham rats (both *P* <0.001). Comparing the disability subgroups, IL-6 expression was significantly increased in only *Pain and disability* rats (90.2 ± 9.9 pg/ml, *P* <0.001) compared to sham rats (35.7 ± 6.8 pg/ml). MCP-1 expression was significantly increased in both *Pain and disability* (152.1 ± 21.9 pg/ml, *P* <0.01) and *Pain alone* (155.4 ± 3.8 pg/ml, *P* <0.01) rats compared to shams (83.1 ± 4.0 pg/ml). These data show a pro-inflammatory response in the DRG after CCI, which is strongest in *Pain and disability* rats.

**Table 2:** Mean cytokine concentration (pg/mL) (±SEM) in the ipsilateral L4-L5 DRG seven days following CCI

Cytokine	Sham (*n* = 5)	Combined CCI (*n* = 14)	*Pain and disability* (*n* = 5)	*Pain and transient disability* (*n* = 4)	*Pain alone* (*n* = 5)
IL-1β	65.3 ± 10.4	98.9 ± 8.8	102.1 ± 16.6	111.1 ± 24.9	86.0 ± 6.1
IL-6	35.7 ± 6.8	82.5 ± 4.7***	90.2 ± 9.9***	78.9 ± 12.4	77.5 ± 2.2
IL-10	255.4 ± 44.3	251.1 ± 18.0	217.2 ± 21.0	310.6 ± 34.2	237.4 ± 31.6
IL-17A	98.6 ± 17.0	89.0 ± 6.6	77.3 ± 5.0	106.7 ± 18.1	86.5 ± 10.6
IL-18	117.6 ± 13.6	108.1 ± 4.9	98.7 ± 6.9	123.5 ± 9.7	105.1 ± 7.5
TNF	30.0 ± 3.2	28.7 ± 1.3	27.4 ± 2.2	30.9 ± 2.8	28.3 ± 2.4
IFN-γ	47.1 ± 10.8	46.7 ± 4.9	42.2 ± 6.0	56.7 ± 13.6	43.1 ± 5.1
MCP-1	83.1 ± 4.0	149.0 ± 7.6***	152.1 ± 21.9**	136.8 ± 7.8	155.4 ± 3.8**

Statistically significant differences compared to sham are indicated by *P <0.05, **P <0.01 and ***P <0.001. All significant differences have been corrected for family-wise error rate with the Benjamini-Hochberg procedure, with a false discovery rate of *q* <0.05

### CCI induces increased cytokine release in the spinal cord, with *Pain and disability* rats showing the highest cytokine levels

Cytokine expression was examined in whole L4-L5 spinal cord segments seven days after CCI (Table 3). IL-1β, IL-6 and MCP-1 were all significantly increased in CCI rats compared to sham rats (*P* <0.001, *P* <0.05 and *P* <0.05, respectively). Within disability subgroups, only IL-1β was increased selectively in *Pain and disability* (143.6 ± 9.3 pg/ml, *P* <0.01) compared to sham rats (88.3 ± 5.8 pg/ml). Therefore, CCI induces a pro-inflammatory response in sciatic nerve recipient segments of the spinal cord, which is more pronounced in the *Pain and disability* group.

**Table 3:** Mean cytokine concentration (pg/mL) (±SEM) in the L4-L5 spinal cord segment seven days following CCI

Cytokine	Sham (*n* = 5)	Combined CCI (*n* = 14)	*Pain and disability* (*n* = 5)	*Pain and transient disability* (*n* = 4)	*Pain alone* (*n* = 5)
IL-1β	88.3 ± 5.8	129.2 ± 5.6***	143.6 ± 9.3**	116.8 ± 6.4	124.6 ± 10.4
IL-6	58.8 ± 12.1	88.6 ± 4.7*	95.5 ± 7.1	92.3 ± 9.8	78.9 ± 8.3
IL-10	467.6 ± 54.3	559.4 ± 23.8	573.6 ± 19.8	579.1 ± 61.4	529.5 ± 50.3
IL-17A	277.5 ± 33.8	356.8 ± 21.4	409.7 ± 32.9	351.0 ± 22.8	308.6 ± 42.7
IL-18	104.0 ± 11.6	116.2 ± 5.5	112.2 ± 6.4	127.8 ± 14.5	110.9 ± 9.3
TNF	59.8 ± 2.9	66.6 ± 1.9	62.1 ± 1.6	70.2 ± 4.3	66.4 ± 3.7
IFN-γ	60.0 ± 11.2	66.44 ± 6.3	53.4 ± 9.0	65.3 ± 15.3	76.0 ± 9.0
MCP-1	111.7 ± 10.3	135.6 ± 3.5*	138.4 ± 4.5	135.9 ± 6.2	132.6 ± 8.4

Statistically significance differences compared to sham are indicated by *P <0.05, **P <0.01 and ***P <0.001. All significant differences have been corrected for family-wise error rate with the Benjamini-Hochberg procedure, with a false discovery rate of *q* <0.05

## Discussion

This study characterised individual differences in the immune response at peripheral and spinal sites following CCI in rats. Whilst all CCI rats have significant infiltration of macrophages and T lymphocytes into both the injury site and the DRG, it is the *Pain and disability* rats that have the greatest number of both cell types. Furthermore, *Pain and disability* rats have select increases in IL-6 and MCP-1 in the sciatic nerve and IL-6 in the DRG. All rats have an increase in IL-1β, IL-6 and MCP-1 in the spinal cord. However, only *Pain and disability* rats have a significant increase in spinal IL-1β. These observations lead us to suggest that individual immune signatures play a significant role in the different behavioural trajectories following CCI, and in some cases may lead to persistent behavioural disabilities.

### Differential behavioural disabilities, despite near identical sensory abnormalities and structural damage after CCI

In keeping with our published studies [35–42, 46], we have established that three entirely different responses in resident-intruder social interactions occur following sciatic nerve CCI, with the *Pain and disability* group displaying a persistent reduction in dominance behaviour. A reduction in dominance behaviour is concomitant with increased non-social behaviour as well as repetitive approach-avoid behaviour, indicative of a heightened “risk-assessment” coupled with an uncertainty of appropriate action [47, 48]. Despite this, all rats display similar mechanical allodynia, again consistent with our previous findings [35, 37].

To determine whether the expression of disability simply reflected differences in the degree of physical damage to the sciatic nerve, we examined the ultrastructure of injured sciatic nerves using transmission electron microscopy in representative animals at the extremes of the resident-intruder behavioural continuum. Rats whose dominance behaviour was completely unaffected by CCI were compared with rats whose dominance behaviours were dramatically reduced by the injury procedure. The overall finding was that the degree of physical damage was not sufficient to explain these differences in social interactions; we found that an equal reduction in myelin thickness 1 mm from the injury site occurs in both *Pain and disability* and *Pain alone* rats. Similarly, an earlier study reported no structural differences 1 cm proximal to the ligatures, whilst closer to the injury (5 mm) there were signs of endoneurial oedema and degeneration of some myelinated fibres ten days after CCI [49]. Wallerian degeneration distal to the injury site is severe, characterised by a profound loss of myelinated fibres and large oedematous spaces [49]. We also observed severe damage at distal sites, and therefore did not proceed with quantitative analysis.

As Wallerian degeneration proceeds, disintegration of the myelin sheath occurs within 1–2 days at the site of nerve injury [50]. Axonal damage can be examined using the structural protein S100, which is preferentially distributed in myelin-forming Schwann cells [51]. Morphological changes and a generalised decrease in the intensity of S100-IR in all CCI rats six days post-injury is indicative of severe demyelination and is consistent with previous studies that examined S100 after nerve injury [52, 53].

ATF3 has long been considered a marker of sensory fibre damage, being rapidly expressed in almost all DRG neurons following axotomy [54, 55]. The number of ATF3 positive neurons in the L4 and L5 DRG of rats after CCI has been reported to be around 20 % of total neurons after 10–14 days [56, 57], which is broadly in keeping with the 10–15 % reported here six days after CCI. There was no significant difference between ATF3 positive neurons in *Pain and disability* rats and those without ongoing disabilities (*Pain alone* and *Pain and transient disability*). However, *Pain and disability* rats had the fewest ATF3 cells, raising the possibility that their injury was slightly less severe even though the reduced Schwann cell staining and myelin thickness do not support this view. In summary, the three disability groups displayed similar levels of mechanical allodynia and were characterised by near identical damage and structural re-organisation in both the injured nerve and DRG. Thus, differences in behavioural disability defined by altered social interactions are unlikely to be caused by any inherent variability in the CCI procedure itself.

### Differential patterns of immune response in the peripheral nervous system

#### Peripheral cytokines

Diffusible immune cytokines and chemokines are released by resident immune (mast cells and macrophages) and glial cells (Schwann and satellite cells), as well as infiltrating immune cells (macrophages, neutrophils, and T lymphocytes) at both the site of injury and the DRG (for detailed review, see [1]). In animal models of neuropathic pain, these cells release the archetypal pro-inflammatory cytokines, IL-1β, IL-6, TNF and IFN-γ [2], as well as the more recently described IL-17 [58, 59] and IL-18 [60, 61]. These cytokines are capable of directly activating, or sensitising, primary afferent nociceptors leading to allodynia and hyperalgesia (for detailed reviews, see [1] and [2]). Furthermore, across several nerve injury models displaying differential sensory abnormalities (CCI, partial sciatic nerve ligation and axotomy), allodynia was shown to correlate with the numbers of ED1, IL-6 and TNF positive cells [62]. Therefore, it is clear that the immune system plays an important role in the development of the sensory-discriminative aspects pain.

Despite the wealth of knowledge surrounding the role of cytokines in mediating sensory abnormalities in models of nerve injury, few have evaluated their role in the expression of affective-motivational disturbances. In the current study, there were robust increases in IL-1β and IFN-γ in the sciatic nerve of all CCI rats. However, given that the *Pain and disability* rats have the highest number of macrophages and T lymphocytes, it is perhaps unsurprising that this subgroup of rats had highest levels of the pro-inflammatory cytokine IL-6 in the sciatic nerve and DRG, since it is produced by both cell types.

A recent study described preexisting individual differences in the peripheral immune system that predicted susceptibility or resilience to repeated social defeat stress. Specifically, susceptible mice had elevated baseline plasma IL-6, monocytes and T lymphocytes; whilst of the cytokines upregulated by stress, IL-6 showed the greatest increase [63]. This study raises the possibility that *Pain and disability* rats have a preexisting elevation of plasma IL-6, as well as circulating monocytes and T lymphocytes, which explains the exaggerated immune response in peripheral tissues after nerve injury and may also render this subgroup susceptible to behavioural disability.

Elevated expression of the chemokine MCP-1 in the sciatic nerve of *Pain and disability* rats may explain recruitment of greatest number of immune cells in this subgroup. MCP-1 is normally released by damaged nerves and its mRNA is upregulated in the DRG neurons after nerve injury [64]. This ultimately leads to the recruitment of macrophages to the site of nerve injury and the DRG, which are essential to phagocytose the dead and dying remnants of damaged nerve fibres. However, MCP-1 release in the peripheral nervous system (PNS) has previously been linked to the development of sensory abnormalities [65]. A surprising finding was that in the sciatic nerve increased levels of MCP-1 were related to the expression of disability.

Although we did not measure the concentration of circulating cytokines, elevated plasma TNF levels have been reported three and seven days after SNI [29]. Clinically, peripheral blood mononuclear cells (PBMCs) collected from chronic pain patients are primed to produce an exaggerated immune response when stimulated with toll-like receptor agonists [66]. Furthermore, patients with painful neuropathy have increased serum expression of TNF and IL-2 (a T cell activator), with the highest levels of TNF found in patients with comorbid depression [67].

#### T lymphocytes

Infiltration of T lymphocytes to the injured sciatic nerve following CCI was first described by Moalem-Taylor and colleagues, peaking 21 days after injury [52]. It was further established that CD4+ helper T lymphocytes of the Th1 subtype enhance pain hypersensitivity, whilst those of the Th2 subtype attenuate sensory abnormalities. Since then IL-17 positive CD4+ T lymphocytes (Th17) cells have also been implicated in contributing to pain hypersensitivity [59], whereas regulatory T lymphocytes (Tregs) have been shown reverse mechanical allodynia in neuropathic animals [68, 69]. A recent study in humans found a shift towards Tregs and away from Th17 cells in the blood of neuropathic pain patients compared to controls [70]. In light of the pre-clinic findings, where Tregs were anti-nociceptive and Th17 pro-nociceptive, the clinical findings were unexpected. However, whether the balance towards anti-inflammatory Tregs in neuropathic pain patients represents an underlying pathophysiological mechanism, or is an epiphenomenon, due to either an attempt to compensate for ongoing inflammation, or immunosuppression through the chronic stress of suffering from chronic pain, remains to be determined. Further, the differences between clinical and pre-clinical findings may in large part be due to the different time-scales of neuropathy, 2–4 weeks in rodents [59, 68, 69] compared to months to years clinically [70].

In the current study, there was no relationship between the number of T lymphocytes and allodynia, although the level of disability was related to their number. Using the widespread marker, TCRαβ, did not allow examination of which T lymphocyte subset (CD8 or CD4 + Th1/Th2/Th17/Treg) predominated in the nerves of *Pain and disability* rats, and this is certainly an important question for future investigations.

To date, we are not aware of any studies that have reported the impact of T lymphocytes on complex behaviours following nerve injury. However, adoptive transfer of Th17 cells promoted depressive-like behaviour as well as reducing social behaviour in mice, effects which could be reversed by depleting Th17 or blocking IL-17A [71]. Interestingly, *Pain and disability* rats had no change in sciatic nerve IL-17A, whereas *Pain and transient disability* and *Pain alone* experienced a significant reduction in IL-17A, suggesting a possible reduction in Th17 cells in the non-disabled groups, which may be related to maintenance of dominance behaviour.

There have been reports of T lymphocyte-dependent coping behaviours in response to significant physical stressors. Chronic restraint stress induces increased serum levels of IL-6 and IL-1β in wild-type mice, whilst mice lacking T-bet, a transcription factor necessary for Th1 differentiation, were protected from these maladaptive responses [72]. More recently, depletion of Treg cells in wild-type mice potentiated depressive-like and anxiety-like behaviours, as well as serum IL-6 and TNF levels following chronic restraint [73]. Furthermore, in Treg-depleted mice, immobility in the forced swim test positively correlated with the levels of peripheral cytokines, but negatively correlated with the levels of 5-HT in the hippocampus and dopamine in the prefrontal cortex [73]. Thus, T lymphocytes have the capacity to modulate depressive-like behaviour, as well as coping responses to physical stressors in mice, raising the possibility of a similar role in the expression of pain and disability in a subgroup of rats.

The elevated T lymphocytes in *Pain and disability* rats are indicative of an enhanced adaptive immune response in this subgroup. This could be in part due to a greater adaptive response to the chromic gut itself, with enhanced sensitivity to the foreign antigens (bovine or ovine intestines) or the chemical treatment (chromium ions or pyrogallol). However, chromic gut is essential for the reliable development of allodynia, as well as spinal cord level central sensitisation, compared to silk sutures that induced neither [74, 75].

Mechanisms for differential adaptive immune responses have been strongly linked with HPA axis activity. We have shown previously that CCI rats from all behavioural groups have elevated plasma corticosterone levels, peaking on day three [41]. It is also known that rats with distinct emotional coping responses (proactive versus reactive) show differences in HPA reactivity and helper T lymphocyte polarisation in response to stressors [76, 77]. Elevated glucocorticoids are thought to be anti-inflammatory, shifting the balance of helper T lymphocytes towards Th2 [78]. A glucocorticoid-mediated mechanism may explain the shift towards Tregs in neuropathic pain patients [70].

The basis of individual differences in the HPA-immune relationship may well find an origin in early life; for example, maternal separation led to a subpopulation of mice developing glucocorticoid resistance of splenic lymphocytes and hence an enhanced pro-inflammatory cytokine response to subsequent immune challenges [79]. It is tempting to speculate that lymphocyte resistance to glucocorticoids develops in rats of the *Pain and disability* group leading to a more pro-inflammatory response (via loss of Th2), resulting in the expression of disability. Further studies are required to investigate this possibility.

### Differential patterns of immune response in the central nervous system

The dorsal horn of the spinal cord contains the first synapse of the nociceptive pathway. Thus, there is a robust activation of astrocytes and microglia [80, 81], as well as infiltration of monocytes and T lymphocytes [82–84] in response to mediators such as ATP and MCP-1 released from damaged and hyperexcitable sensory nerve fibres in the spinal cord following nerve injury. Activation of these cell types induces release of cytokines such as IL-1β, IL-6 and TNF in high concentrations [85]. This creates a positive loop of activation, since cytokines further exacerbate glial activation and thereby potentiate their own release. Cytokines and chemokines, such as MCP-1, can act on neurons directly, modulating ascending nociceptive pathways to exacerbate sensory abnormalities [1, 2] or potentially modulate affective-motivational signalling. Indeed, the role of neuro-immune interactions in driving *Pain and disability* has been proposed in our recent study examining gene transcription in the spinal cord after CCI [43]. Of the 35 genes that were selectively regulated in *Pain and disability* rats, approximately two-thirds (65 %) were genes for protein involved in neurotransmission and inflammation (compared to only 34 % in rats without disability). In particular, selective increases in mRNA for complement component 3, ciliary neurotrophic factor (an IL-6-like cytokine), prostaglandin receptor F and P2X_2_ receptors were discovered in the spinal cord of *Pain and disability* rats, indicating an exaggerated and unique immune signature in these rats. These findings fit well with our current observations, where *Pain and disability* rats had a selective increase in spinal cord IL-1β, whilst the expression levels of IL-6 and MCP-1 were also highest in this subgroup of rats. These immune mediators may well selectively modulate ascending pathways specific to the expression of disability.

Several studies have reported that GFAP (indicative of astrocyte activation) and CD11b expression are increased in the PAG following nerve injury [23, 24, 39]. Of particular relevance here, *Pain and disability* rats have elevated GFAP in lateral and ventrolateral columns of the PAG [39], which receive ascending projections from the spinal cord and nucleus of the solitary tract, and are critical in the regulation of specific behavioural coping strategies [86]. Therefore, it is possible that differences in the neuro-immune signature in the spinal cords of *Pain and disability* rats selectively modulate ascending spinal-mesencephalic projections leading to the specific pattern of GFAP expression seen in this subgroup, consequently contributing to the expression of disability.

## Conclusions

This study is the first to characterise individual differences in immune response to CCI at peripheral and spinal sites, despite the same degree of damage and sensory abnormalities. These differential patterns of immune response correlate with the degree of disability in social interactions, with the *Pain and disability* subgroup displaying a distinct pro-inflammatory signature. Two recent clinical studies highlight the importance of individual differences in the immune system in modulating functional recovery from injury. Firstly, using mass spectrometry Gaudilliere and colleagues demonstrated recovery from hip arthroplasty correlates with a specific single-cell immune signature in monocytes [87]. Secondly, Schistad and colleagues demonstrated that high serum IL-6 is associated with poor long-term (one year) recovery from lumbar radicular pain [88]. Thus, distinct immune signatures in individual rats at multiple levels of the neuraxis in response to traumatic nerve injury likely influence the trajectory of recovery from CCI, particularly the expression of behavioural disabilities. Simply put, an individual’s immune signature may on one hand lead to functional recovery or on the other to the development of persistent pain accompanied by debilitating changes in affective-motivational state.

## Abbreviations

ATF3: Activating transcription factor 3
CCI: Chronic constriction injury
CNS: Central nervous system
DRG: Dorsal root ganglia
GFAP: Glial fibrillary protein
HPA: Hypothalamic-pituitary-adrenal
IDO: Indoleamine 2,3-dioxgenase
IFN-γ: Interferon-γ
IL-1β: Interleukin-1β
IL-6: Interleukin-6
IL-10: Interleukin-10
IL-17A: Interleukin-17A
IL-18: Interleukin-18
IR: Immunoreactivity
MCP-1: Monocyte chemoattractant protein-1
NAcc: Nucleus accumbens
PAG: Periaqueductal gray
PBMCs: Peripheral blood mononuclear cells
PNS: Peripheral nervous system
SNI: Spared nerve injury
TNF: Tumor necrosis factor
Tregs: Regulatory T lymphocytes
